# Semantic memory for actions as assessed by the Kissing and Dancing Test: Education and age effects in cognitively healthy individuals

**DOI:** 10.1590/S1980-57642014DN83000004

**Published:** 2014

**Authors:** Roberta Roque Baradel, Henrique Salmazo da Silva, Jaqueline Geraldin Estequi, Maria Alice de Mattos Pimenta Parente, João Ricardo Sato, Maria Teresa Carthery-Goulart

**Affiliations:** 1 UFABC – Universidade Federal do ABC. Center of Mathematics, Computation and Cognition. Neuroscience and Cognition. Post-graduation. Language and Cognition Research Group (GELC-UFABC), Santo André, SP, Brazil; 2 Behavioural and Cognitive Neurology Unit, University of São Paulo, School of Medicine, São Paulo, Brazil.

**Keywords:** cognition, language, memory, neuropsychological tests, age, schooling

## Abstract

Action semantics is a relevant part of cognitive-linguistic assessment and the "Kissing and Dancing Test" (KDT) has been used extensively for this purpose, evidencing clinical distinctions among brain-damaged patients. To enhance its use, reference values are necessary, especially for populations with heterogeneous educational levels and socioeconomic backgrounds.

**Objective:**

To analyze the effects of schooling and age on the KDT in cognitively unimpaired individuals.

**Methods:**

The KDT was applied to seventy-four healthy subjects. Sociodemographic factors were investigated through correlational and between-group analyses. Reference values according to age and schooling were provided.

**Results:**

KDT performance correlated significantly with schooling (r=0.757, p<0.01), age (r=–0.496, p<0.01) and socioeconomic status (r=0.418 p<0.01) but these variables were intercorrelated. Correlation with schooling and age remained significant when controlling for age and socioeconomic status (r=0.530, p<0.01), and for schooling (–0.305,<0.01), respectively. When controlling for schooling, correlation between socioeconomic status and KDT was not significant (p=0.164). Between-group analyses revealed no age effects. Significant differences were found in performance according to educational level. Scores below 39/52 and below 47/52 (percentile 25) for individuals with 8 or less years of schooling and for individuals with 9 or more years of schooling, respectively, seem suggestive of an impairment in Action Semantics Processing and shall be further investigated.

**Conclusion:**

KDT performance was influenced both by age and schooling, indicating the need to consider these demographic features as covariates when analyzing performance on the test and to adjust cut-off scores according to these demographic characteristics in clinical practice.

## Abstract (pt)

Processamento semântico verbal é uma parte relevante da avaliação linguístico-cognitiva e o KDT tem sido amplamente utilizado para tal finalidade, evidenciando diferenças clínicas entre pacientes com danos cerebrais. Para aumentar sua utilização, valores de referência são necessários, especialmente para populações com origens socioeconômicas e níveis de escolaridade heterogêneos.

**Objetivo:**

Analisar efeitos da escolaridade e idade no KDT em indivíduos cognitivamente intactos.

**Métodos:**

O KDT foi aplicado em 74 indivíduos saudáveis. Fatores sociodemográficos foram investigados por correlação e análises entre grupos. Valores de referência foram fornecidos por idade e escolaridade.

**Resultados:**

O desempenho do KDT correlaciona-se significativamente à escolaridade (r=0.757, p<0.01), à idade (r=–0.496, p<0.01) e ao nível socioeconômico (r=0.418, p<0.01), variáveis que se mostraram intercorrelacionadas. Correlações com a escolaridade e idade permaneceram significativas quando controladas a idade e nível socioeconômico (r=0.530, p<0.01) e a escolaridade (r=- 0.305,<0.01), respectivamente. Com controle do parâmetro da escolaridade, não houve correlação significativa entre o nível socioeconômico e o KDT (p=0.164). As análises entre grupos não revelaram os efeitos da idade. Encontraram-se diferenças significativas no desempenho de acordo com escolaridade. Escores abaixo do percentil 25 (39/52 -indivíduos com até 8 anos de escolaridade e 47/52- indivíduos com 9 ou mais anos de escolaridade) são sugestivos de dificuldades no processamento semântico de ações, que devem ser melhor investigadas.

**Conclusão:**

O desempenho do KDT foi influenciado por idade e escolaridade, o que reflete a necessidade de se considerar estes fatores como covariantes e, na prática clínica, usar diferentes pontos de corte, considerando essas características demográficas.

## INTRODUCTION

According to Tulving,^1^ Semantic memory is a cognitive system that allows you to build a collection of information, knowledge, skills and other concepts related to particular experiences. This type of memory has been the subject of several studies in the field of Cognitive Neuropsychology, involving populations with and without neurological disorders.^2^ In particular, the dissociation in the semantic processing of nouns vs verbs has generated important discussions about the neural substrates of semantic memory and it has been suggested they may constitute distinct processes.^3-5^ In a recent review of the literature, Crepaldi et al.^6^ observed a trend of more areas of brain activation for verbs compared to nouns, suggesting that verbs have higher semantic complexity. In addition, both Crepaldi et al.^6^ and Vigliocco et al.,^7^ showed that tasks requiring more semantic processing accentuate the differences between these two grammatical classes and that verbs tend to be morphologically more complex than nouns in many languages.

Thus, as noted in the postulates mentioned above, the dissociation between nouns and verbs prompts and justifies more extensive research to discuss and clarify conflicting results in this area.^6,7^ This would require the pursuit of more descriptive data,^8^ aiming at "an alternative route of investigation in order to deepen theories of dissociation between nouns and verbs".^8^

Although there are many tools for assessing semantic memory in adults, in relation to the sociolinguistic and cultural context of Brazilian Portuguese, there is a dearth of adapted tests for assessing the cognitive processes involved in the comprehension and use of verbs, especially considering criteria such as age of acquisition, familiarity, frequency, suitability, and visual complexity.^9^

In this scenario, one of the most commonly used tests for investigating action/verb semantics is the Kissing and Dancing Test (KDT),^10^ created to examine the recognition and semantic association of actions to explore neurological processes involved in the use of semantic memory. The test consists of 156 illustrations in black and white, depicting different actions and divided into 52 cards, similar in design, size and spatial distribution. Each card has at the top and center, an illustration representing a verb, followed by two more verbs, one which is directly associated with the target and another that is a distractor, i.e. a verb with no direct relationship with the target. (For examples, see^11^).

The KDT - similar to another test which is restricted to nouns (Pyramids and Palm trees Test)^12^ - has been an important research methodology for the study of verb/noun dissociations, especially in contexts where the participant presents with motor limitations such as severe dysarthria and it is only possible to obtain responses by asking the subject to point at stimuli. Moreover, its picture version (KDT also has a version comprised of written words), does not require reading and may also help investigations conducted in populations with heterogeneous educational backgrounds, including illiterate individuals.

In our population, performance on the KDT has been recently investigated in a sample of Brazilian highly educated healthy young individuals^11^ and the total score obtained was lower than that found with English participants, possibly reflecting cultural differences in processing the pictorial stimuli of the KDT. The authors illustrated the use of the test in two patients with dementia (semantic dementia and the behavioral variant of frontotemporal dementia FTDbv) and observed the predicted effects with a more marked impairment for verbs in FTDbv (compared to nouns as assessed by the PPT) and the opposite pattern in SD. Reference values for young and highly educated individuals were suggested but effects of age and schooling could not be investigated due to the characteristics of the sample.

The relationship between semantic memory, aging and education is a complex issue that has not been sufficiently investigated in the field of action semantics. In a recent study, Verhaegen & Poncelet^13^ investigated naming and semantic abilities across the adult lifespan and reported changes in accessing semantic memory (latency measures) or the lexicon (naming) in individuals aged 50 and over. In individuals aged 70 years or older, results suggested degraded semantic representations with changes in accuracy rates. However, the study in question focused on nouns and did not include low educated individuals. Do these changes also occur with stimuli depicting actions? How do low educated individuals perform on these tasks?

In an effort to further the investigation of these questions, the current study sought to characterize the performance of a sample of cognitively healthy individuals on the KDT, focusing on the impact of aging and education in this test. In addition, we present reference values that may be useful to guide clinical practice.

## METHODS

**Participants.** The KDT was applied on a one-to-one basis to seventy-four cognitively healthy adults in a silent room, using conventional triad presentation, on A^4^ cards. The sample was obtained by convenience in community centers for the elderly and Universities in the metropolitan region of São Paulo. The following instruction was given: *"Here we have three drawings. You need to decide which of these on the bottom match the one on the top. Is it this one or the other"?* One point was given for each correct answer. All participants were functionally independent and had normal global cognitive functioning, as assessed by the Mini Mental State Examination (MMSE) using schooling-adjusted scores proposed by Brucki et al.^14^ Functional Independence was assessed with a semi-structured interview, applied to the participants aged 60 years or older only (self-report), investigating ten instrumental activities of daily-living. None of those participants reported difficulties independently performing these activities. Their performance was also within the normal range on the Clock Drawing Test^15^ and the Brief Cognitive Battery-Edu.^16^

Participants were divided into 3 age groups (Group 1: 20-39, Group 2: 40-59 and Group 3: 60 and older) and 3 categories of education (Group 1: up to 4 years, Group 2: 5-8 years and Group 3: 9 years and over)-(Table 1). Socioeconomic status was evaluated by the CCEB questionnaire (Critério de Classificação Econômica do Brasil) developed by the Associação Brasileira de Empresas de Pesquisa (ABEP)^17^ that classifies individuals into seven classes A1 (42-46), A2 (35-41), B1 (29-34), B2 (23-28), C (18-22), D (14-17) and E (0-7).

**Table 1:** Clinical and demographic features of the sample according to age and level of schooling.

		Sex Nº women	Age Mean (SD)	Education Mean (SD)	MMSE Mean (SD)	CDT Mean (SD)	BCB Delayed Recall Mean (SD)
Age groups	20 |- 39 (n=19)	10	24.4(6.8)	14 (1.9)	28.84 (±1.26)	8.16 (±2.61)	9.37 (±0.89)
	40 |- 59 (n=32)	27	50.2 (5.1)	6.9 (4)	26.94 (±2.52)	8.50 (±1.50)	8.75 (±1.34)
	60 |- 82 (n=23)	17	68.4(5.9)	8.6 (5)	27.61 (±1.73)	7.67 (±2.35)	8.52 (±1.50)
Schooling groups	0 |- 4 (n=17)	15	60.4 (10.4)	3.2 (1.3)	25.35 (±2.73)	7.47 (±2.29)	8.65 (±1.41)
	5 |- 8 (n=19)	6	54.4 (10.3)	6.2 (1.2)	27.58 (±1.46)	8.61 (±1.24)	8.37 (±1.53)
	9 |- 17 (n=39)	23	41.7 (19.6)	13.4 (2.4)	28.68 (±1.12)	8.20 (±2.37)	9.16 (±1.10)
Total (n=74)		54	49.2 (17.6)	9.2 (4.8)	27.60 (±1.70)	8.15 (±2.13)	8.90 (±1.32 )

SD: standard deviation; MMSE: Mini-Mental State Examination; CDT: Clock Drawing Test; BCB Delayed Recall: Brief Cognitive Battery-Edu, delayed recall task.

This study is part of a research project about semantic memory that was approved by the Ethics Committee of the University of São Paulo City (UNICID) under nº CAAE 4461.0.000.107-10.

**Statistical analysis.** The analyses were performed with IBM SPSS 20 (www.ibm.com/software/analytics/spss). We assessed the data adherence to a Gaussian distribution by using the Shapiro-Wilk test and observed that the variables significantly deviated from this distribution (p<0.01). Therefore, nonparametric tests were employed in the subsequent analyses.

Spearman correlation analyses between KDT total scores, age (in years), education (in number of years of formal education) and socioeconomic status (total score on CCEB questionnaire) were conducted. Partial correlations were also calculated in order to evaluate the relationship between KDT and each of these variables independently.

The Kruskal-Wallis rank test and the Mann-Whitney test were used to conduct between-group analyses in order to further explore age and education effects.

The significance level for the analyses was set at 1% (considering Bonferroni corrections for multiple comparisons).

Based on the results of the between-group analysis, reference values for the use of KDT (mean, standard deviation, range and percentiles (10-25-50-75-90) were presented and stimuli that yielded more errors in the sample described.

## RESULTS

**Subjects.** Clinical and demographic features of the sample are presented in Table 1. Seventy-one subjects answered the CCEB questionnaire in full (three subjects provided incomplete information and their scores could not be included in the analyses). The scores ranged between 12 and 40, mean score was 24.37 and Standard Deviation was 6.5. Most individuals in the sample were classified into level C (n=22), followed by B2 (n=21), B1 (n=10), D (n=9), A2 (n=8) and E (n=1). Four individuals were left-handed, and five individuals reported speaking one or two languages other than Portuguese.

**Correlation analyses.** Significant positive correlations were found between education and KDT (r=0.757, p<0.01) and socioeconomic status and KDT (r=0.418, p<0.01). A negative significant correlation between KDT and age (r=–0.496, p<0.01) was observed. Education was significantly correlated with Age (r=–0.409, p<0.01), as well as with Socioeconomic Status (r=0.416, p<0.01). The correlation between KDT and education remained significant after controlling for Age and Socioeconomic Status (partial correlation) (r=0.530 p<0.01). The correlation between age and KDT also remained significant when controlling for Education (r=–0.305 p<0.01). However, correlation with Socioeconomic level was not significant after controlling for Education (r=0.168, p=0.164).

### Between-group analyses

*Age effects.* As the younger group in our sample comprised only highly educated individuals, we conducted two different between-group analyses to investigate age effects. In the first analysis, only subjects from Group 3 (individuals with the highest education in the sample) were included. In the second, the younger age group (Group 1) was excluded and age effects evaluated in Groups 2 and 3 (aged 40 and over) and that presented subjects in the three education groups (Table 2).

**Table 2:** Performance on KDT according to age group.

**Individuals with 9 or more years of schooling aged 20 or over**					
	Age groups	Mean	SD	Range	p
KDT	1 (N=19)	49.68	1.974	45-52	.054
	2 (N=9)	46.78	3.114	42-52	
	3 (N=10)	47.50	3.837	41-52	
Age	1 (N=19)	24.37	6.841	18-39	<0.01
	2 (N=9)	49.89	5.510	43-57	
	3 (N=10)	67.30	6.290	60-78	
Education	1 (N=19)	13.95	1.900	11-17	.163
	2 (N=9)	12.11	2.667	09-16	
	3 (N=10)	13.60	2.836	10-17	
Socioeconomic	1 (N=19)	25.53	6.345	14-37	.153
	2 (N=9)	26.11	5.819	19-39	
	3 (N=10)	30.78	7.242	21-39	
**Individuals with 1-17 years of schooling aged 40 or over**					
	**Age groups**	**Mean**	**SD**	**Range**	**P**
KDT	2 (N=32)	43.47	4.325	36-52	.596
	3 (N=23)	44.13	5.155	35-52	
Age	2 (N=32)	50.22	5.123	40-57	<0.01
	3 (N=23)	68.43	5.968	60-82	
Education	2 (N=32)	6.88	4.030	0-16	.315
	3 (N=23)	8.57	5.017	2-17	
Socioeconomic	2 (N=32)	27.17	5.246	12-39	.860
	3 (N=23)	25.00	8.153	14-40	

SD: standard deviation; KDT: Kissing and Dancing Test; Socioeconomic: Socioeconomic level according to CCEB.

As assessed by the Kruskal-Wallis test, no significant age differences were observed in the performance of KDT among highly educated individuals (p=0.054). However, visual inspection of the data shows higher scores and lower standard deviation values in the younger group.

In the second analysis (excluding the younger group), the Mann-Whitney test revealed no significant differences between the younger (40-59 years old) and older (60 - 82) adult groups in KDT scores (p=.596).

*Education effects.* For this analysis, only individuals aged 40 and over were included, since among the younger subjects there were only highly educated individuals. The Kruskal-Wallis test showed a significant difference in KDT performance in the three schooling groups. Mann-Whitney analyses showed that groups 1 and 2 did not differ significantly in performance (p=0.207) and Group 3 performed significantly better than Groups 1 (p<0.01) and 2 (p<0.01). (Table 3)

**Table 3:** Performance on the KDT according to schooling croup.

	Schooling groups	Mean	SD	Range	P
KDT	1 (N=17)	40.76	4.381	45-52	<0.01*
	2 (N=19)	43.00	3.859	42-52	
	3(N=19)	47.16	3.436	41-52	
Age	1 (N=17)	60.35	10.440	18-39	0.175
	2 (N=19)	54.37	10.259	43-57	
	3(N=19)	59.05	10.633	60-78	
Education	1 (N=17)	3.18	1.334	11-17	0.01*
	2 (N=19)	6.21	1.182	9-16	
	3(N=19)	12.89	2.787	10-17	
Socioeconomic	1 (N=17)	21.73	6.100	14-37	<0.01*
	2 (N=19)	21.42	4.525	19-39	
	3 (N=19)	28.44	6.810	21-39	

SD: standard deviation; KDT: Kissing and Dancing Test; Socioeconomic: Socioeconomic level according to CCEB.

* Significant differences between Group 3 and Groups 1 and 2 (Mann-Whitney test).

Based on the results of the between-group analyses, separate reference values for Group 3 (38 subjects) and for Groups 1 and 2 (36 cases) are shown (Table 4).

**Table 4:** Reference values for the use of KDT according to education.

Education	Mean (SD)	Range	10 Percentile	25 Percentile	50 Percentile	75 Percentile	90 Percentile
≤8 (N=36)	41.94 (4.2)	35-50	36	39	43	36	47
≥9 (N=38)	48.42 (3.04)	41-52	43	47	49	51	52

SD: standard deviation.

*Error analysis.* Among the 52 stimuli, 15 yielded errors in more than 20% of the sample (Table 5). These items were removed and correlation and between-group analyses repeated, yielding the same significant correlations (Age, r=-.278 p<0.01and Education, r=.636 p<0.01) and differences between schooling groups.

**Table 5:** KDT stimuli that yielded errors in 20% or more of the individuals in the sample.

Stimuli	% of correct responses	Stimuli	% of correct responses	Stimuli	% of correct responses
8 – Painting	75.7	18 – Riding	75.7	38 – Ringing	77
10 – Drilling	74.3	22 – Taking clothes	78.4	39 – Snowing	78.4
12 – Fighting	67.6	23 – Skiing	67.6	40 – Roaring	79.7
13 – Smiling	63.5	24 – Arguing	62.2	46 – Knocking	75.7
15 – Shutting	58.1	26 – Buying	64.9	48 – Watering	73

## DISCUSSION

This study was aimed at exploring the impact of age and education (measured as years of formal schooling) in performance on a pictorial test of semantic memory, the KDT. In our study, performance of cognitively unimpaired subjects was associated with age and education and, as observed in other similar research, these variables were correlated, since low education is more common among the elderly.^18-20^

Changes in semantic memory processing may be the first clinical symptom of some neurodegenerative diseases^21,22^ yet there are few available neuropsychological tools to assess these in populations with heterogeneous socioeconomic and educational backgrounds. More specifically, the KDT evaluates Action Semantics, an aspect of cognition that has received considerable attention in the literature, both regarding the scientific debate on the neural representation of actions/ verbs^23-25^ and also concerning clinical characteristics of particular disorders in which double dissociations in noun/verb and/or object/action semantic processing have been described.^6,7,26-28^

In our study, KDT performance was more influenced by education than by age and socioeconomic factors. In fact, the impact of socioeconomic class was no longer significant when education was controlled. The correlation between age and KDT was still significant when controlling for education, corroborating findings of previous studies using similar tasks.^18,20^

In order to investigate these effects further, we conducted between-group analyses, that revealed no significant differences among the three age groups defined, except for a numerical advantage by younger adults in the task, observed only among individuals with high education. This raises the question as to whether changes in semantic memory tasks may occur early during the adult life-span and if so, what their nature might be. Normal aging is usually described in terms of gains in the ability of using well-established knowledge (crystallized intelligence) which contrasts with losses in skills involving learning of new tasks (fluid intelligence).^28,29^ However, regarding noun/object semantics, a recent study^13^ revealed modifications in the ability to access semantic information in individuals in their 50s and changes in accuracy rates in subjects older than 70 years. Our study addressed only accuracy rates, which appeared to be slightly lower in older individuals. One possible explanation may be related to cognitive slowing, which would affect quality of performance in a more general manner by reducing the quantity of available information processed simultaneously. This would interfere in attention, memory, judgment capacity and decision-making, even in activities that do not require speed.^30^ This hypothesis requires further investigation in studies involving larger samples and including young individuals with more varied educational levels. In our study, we were unable to enroll low educated individuals in the younger age range because our sample was recruited by convenience and consisted of individuals working/studying at the recruitment centers. Schooling was higher among the individuals in the youngest group, ranging from 9-17 years.

Schooling plays an important role in performance both when examining complex functions that involve verbal reasoning^31^ and in simple screening tasks, such as the Mini-Mental State Exam^32^ as well as on batteries specially developed for the evaluation of individuals with low education, such as the Brief Cognitive BatteryEdu.^16^ The influence of education in cognitive-linguistic assessment is a highly relevant question, since language mediates the examination of other cognitive functions.

Two previous studies found an impact of education on performance of a similar task but involving nouns instead of verbs (PPT).^18,20^ Their results and now ours, demonstrate that even on tasks requiring no reading or verbal output, education plays a role and should be considered a covariate of performance.

In our study, the two groups with lower education did not differ in KDT performance, with significant changes being detected only when comparing these groups to individuals with 9 or more years of schooling. Our results corroborate the findings of Radanovic et al.^33^ who found no schooling effects for the Brazilian population with over 8 years of schooling on a comprehensive language test. It also confirms the reference values proposed by Mansur et al.^11^ for individuals with high education.

To our knowledge, there is only one previous study that reported KDT performance in individuals with low education.^34^ The cited study was conducted with Chinese participants and the authors replaced stimuli on which 20% or more individuals failed in their sample. They found no influence of education in the study, but the analyses were undertaken after replacing the problematic stimuli, which they considered inappropriate for the population. It is important to mention that KDT has not been adapted for use in our population and some of the pictures may give rise to errors due to cultural aspects. In the present study, even when removing the cards that caused more errors, schooling and, to a lesser degree age, remained significant covariates of performance. Moreover, when comparing our results to those obtained in the study of Mansur et al.,^11^ only five out of fifteen boards coincided among the ones that yielded more errors (items 8,^13,15,23^ and 48). This inconsistency of performance among individuals needs to be analyzed further in order to decide whether items should be replaced or removed.

As reported for other measures addressed in previous studies,^35^ we observed higher variability in performance among individuals with low education. This may indicate that education homogenizes mental organization together with other related factors of cognitive reserve, such as reading and writing habits.^35,36^

Although here we describe reference values for the KDT according to education, since they were derived from a small sample they may be used only as guides for clinical practice, while normative data for the test is not available for low-educated individuals. Another limitation is that few men were recruited, and therefore the influence of sex was not addressed.

Finally, it is important to consider the need for developing new tools to assess semantic memory processing, adapted to the context and parameters of Neuropsychology.^37^ The methodology for developing KDT was not fully described in the original publication^10^ and more specifically, the parameters used in the selection and organization of images (types of verbs, frequency, familiarity, imageability, visual complexity criteria among others). Cultural factors related to graphical presentation were mentioned and possible factors affecting the performance of normal subjects on the KDT.^11^ Santaella, Nöth^38^ in the field of imaging studies in Semiotics, claimed that the process of assigning meaning or determining a referent from a picture is not as simple as it sounds, since pictures drawn by hand are an artisanal process that depend on the ability of an individual to "shape the visible." In this sense, Novaes – Pinto^39^ believes that the occurrence of several figures being "poorly designed" may interfere in semantic memory tasks, even in non-aphasic subjects. These psycholinguistic variables are known to play a role in action/verb semantic tasks and should be addressed appropriately.

## References

[1] Tulving E, Tulving E, Donaldson W (1972). Episodic walks semantic memory. Organization of Memory.

[2] Hiliis A, Chapey R (2008). Cognitive neuropsychological approaches to rehabilitation of
language disorders: introduction. Language intervention strategies in aphasia and related neurogenic
communication disorders.

[3] Caramazza A, Hillis A (1991). Lexical organization of nouns and verbs in the
brain. Nature.

[4] Damasio AR, Tranel D (1993). Nouns and verbs are retrieved with differently distributed neural
systems. Proc Natl Acad Sci USA.

[5] Daniele A, Giustolisi G, Silveri MC, Colosimo C, Gainotti G (1994). Evidence for a possible neuroanatomical basis for lexical
processing of nouns and verbs. Neuropsychologia.

[6] Crepaldi D, Berlingeri M, Paulesu E, Luzzati C (2011). A place for nouns and a place for verbs? A critical review of
neurocognitive data on grammatical class effects. Brain Lang.

[7] Vigliocco G, Vinson DP, Druks J, Barber H, Cappa S (2011). Nouns and verbs in the brain: A review of behavioral,
electrophysiological, neuropsychological and imaging studies. Neuroscience and Biobehavioral Reviews.

[8] Mätzig S, Druks J, Masterson J, Vigliocco G (2009). Noun and verb differences in picture naming: past studies and new
evidence. Cortex.

[9] Spezzano L.C (2012). Study skill naming objects and verbs - analysis of error types.

[10] Bak TH, Hodges JR (2003). Kissing and Dancing - a test to distinguish the lexical and
conceptual contributions to noun / verb and action / object dissociation.
Preliminary results in patients with frontotemporal dementia. J Neuroling.

[11] Mansur L, Goulart MTC, Bahia VS, Bak TH, Nitrini R (2013). Semantic Memory: nouns and action verbs in cognitively unimpaired
individuals and frontotemporal lobar degeneration. Dement Neuropsychol.

[12] Howard D, Patterson K (1992). The Pyramids and Palm Trees Test: A test for semantic accessory from
words and pictures.

[13] Verhaegen C, Poncelet M (2013). Changes in naming and semantic abilities with aging from 50 to 90
years. J Int Neuropsychol Soc.

[14] Brucki SM, Nitrini R, Caramelli P, Bertolucci PH, Okamoto IH (2003). Sugestões para o uso do Mini-Exame do Estado Mental no
Brasil. Arq Neuropsiquiatr.

[15] Sunderland T, Hill JL, Mellow AM (1989). Clock drawing in Alzheimer's disease: a novel measure of dementia
severity. J Am Geriatric Soc.

[16] Nitrini R, Lefrèvre BH, Mathias SC (1994). Testes neuropsicológicos de aplicação
simples para o diagnóstico de demência. Arq Neuropsiquiatr.

[17] Associação Brasileira de Empresas de Pesquisa (2003). Critério de classificação econômica -
Brasil.

[18] Gudayol-Ferré E, Lara JP, Herrera-Guzman I (2008). Semantic memory as assessed by the Pyramids and PalmTrees test:
the impact of sociodemographic factors in a Spanish-speaking
population. J Int Neuropsychol Soc.

[19] Carvalho SA, Barreto SM, Guerra HL, Gama ACC (2009). Oral language comprehension assessment among elderly: a
population based study in Brazil. Prev Med.

[20] Gamboz N, Coluccia E, Iavarone A, Brandimonte MA (2009). Normative data for the Pyramids and Palm Trees test in the
elderly Italian population. Neurol Sci.

[21] Gorno-Tempini ML, Dronkers NF, Rankin KP (2004). Cognition and anatomy in three variants of primary progressive
aphasia. Ann Neurol.

[22] Carthery-Goulart MT, Knibb JA (2012). Semantic dementia versus nonfluent progressive aphasia:
neuropsychological characterization and differentiation. Alzheimer Dis Assoc Disord.

[23] Pulvermüller F, Shtyrov Y, Ilmoniemi R (2005). Brain signatures of meaning access in action word
recognition. J Cog Neurosci.

[24] Kemmerer D, Castillo JG, Talavage T, Patterson S, Wiley C (2008). Neuroanatomical distribution of five semantic components of
verbs: evidence from fMRI. Brain Lang.

[25] Silva HS, Machado J, Cravo A (2014). Parente MAMP, Carthery-Goulart MT. Action-Verb processing:
debates in neuroimagins and the contribuition of studies in patients with
Parkinson's disease. Dement Neuropsychol.

[26] Bak TH, Hodges JR (2004). The effects of motor neuron disease on language: Further
evidence. Brain Lang.

[27] Bak T, Yancopoulou D, Nestor PJ (2006). Clinical, imaging and pathological correlates of a hereditary
deficit in verb and action. Brain.

[28] Parente MAMP, Parente Maria Alice de Mattos Pimenta (2006). Compreensão da linguagem e envelhecimento. Cognição e envelhecimento.

[29] de Oliveira MO, Nitrini R, Yassuda MS, Brucki SMD (2014). Vocabulary is an Appropriate Measure of Premorbid Intelligence in
a Sample with Heterogeneous Educational Level in Brazil. Behav Neurol.

[30] Salthouse TA (1996). The processing-speed theory of adult age differences in
cognition. Psychol Rev.

[31] Kristensen CH, Parente MAMP, Kaszniak AW, Caminha RM (2005). Transtorno de Estresse Pós-Traumático:
Critérios diagnósticos, prevalência e
avaliação. Transtornos do Estresse Pós-Traumático (TEPT): da
neurobiologia à terapia cognitiva.

[32] Brucki SM, Nitrini R, Caramelli P, Bertolucci PH, Okamoto IH (2003). Suggestions for utilization of the mini mental state examination
in Brazil. Arq Neuropsiquiatr.

[33] Radanovic M, Mansur LL, Scaff M (2004). Normative data for the Brazilian population in the Boston
Diagnostic Aphasia Examination: influence of schooling. Braz J Med Biol Res.

[34] Gui Q, He C, Wen X, Song l, Han Z, BI Y (2013). Adapting the Pyramids and Palm Trees Test and the Kissing and
Dancing Test and Developing other semantic tests for the Chinese
population. Appl Psycholing.

[35] Bertolucci PHF, Brucki SMD, Campacci SR, Juliano Y (1994). O Mini-Exame do Estado Mental em uma população
geral: impacto da escolaridade. Arq Neuropsiquiatr.

[36] Pawlowski J, Rodrigues JC, Martins SO, Brondani R, Chaves M, Fonseca RP, Bandeira DR (2013). Avaliação Neuropsicológica Breve de Adultos
pós-Acidente Vascular Cerebral em Hemisfério
Esquerdo. Av Psicol Latinoam.

[37] Fonseca RP, Zimmermann N, Pawlowski J, Fukushi SS (2012). Métodos em avaliação neuropsiológica:
pressupostos gerais, neurocognitivos, neuropsicolinguísticos e
psicométricos. Métodos em psicobiologia, neurociências e
comportamento.

[38] Santaella l, Nöth W (1997). Imagem, cognição, semiótica, mídia.

[39] Novaes-Pinto R (1999). A contribuição do estudo discursivo para uma
análise crítica das categorias clínicas.

